# Evaluating eating behavior treatments by FDA standards

**DOI:** 10.3389/fpsyg.2013.01009

**Published:** 2014-01-03

**Authors:** A. Janet Tomiyama, Britt Ahlstrom, Traci Mann

**Affiliations:** ^1^Department of Psychology, University of CaliforniaLos Angeles, Los Angeles, CA, USA; ^2^Department of Psychology, University of MinnesotaMinneapolis, MN, USA

**Keywords:** FDA, dieting, diets, behavioral treatments, interventions, weight, obesity, eating behavior

## Abstract

Behavioral treatments for obesity are not evaluated by the same criteria as pharmaceutical drugs, even though treatments such as low-calorie dieting are widely prescribed, require patients’ time and investment, and may have risks. The Food and Drug Administration (FDA) has a procedure for evaluating drugs, in which drugmakers must answer the following questions: (1) Is the treatment safe? (2) How dangerous is the condition the intervention is treating? (3) Is the treatment effective? (4) Is the treatment safe and effective for large numbers of people? We argue that using this framework to evaluate behavioral interventions could help identify unanswered research questions on their efficacy and effectiveness, and we use the example of low-calorie dieting to illustrate how FDA criteria might be applied in the context of behavioral medicine.

## INTRODUCTION

Obesity rates in America have more than doubled in the last 30 years, and currently one third of adults are considered obese, and another third are considered overweight (Flegal et al., 2012). Both the increase in obesity levels and the prevalence of obesity are of great concern to our government, medical community, and the public, and significant efforts to prevent and treat obesity are being made. The National Institutes of Health spent $830 million on obesity research in fiscal year 2011. Unlike many diseases, obesity is notable in that there is no gold-standard pharmacological intervention. Most treatment approaches to obesity involve some form of behavior change, and most involve restrictive dieting.

Behavioral interventions, however, are not evaluated according to the same criteria as pharmaceutical drugs. In this article, we argue that examining behavioral interventions using the criteria of the Food and Drug Administration (FDA) is a useful exercise for identifying unanswered research questions on the efficacy and effectiveness of behavioral treatments. We use dieting, or the restriction of caloric intake for the purpose of weight loss, as our exemplar, and outline how researchers might benefit from applying the standard criteria used for evaluating drugs.

The goal of governmental agencies like the FDA is to protect public health and promote the use of safer and more effective treatments. Although behavioral interventions are not regulated by the FDA, behavior can affect health profoundly. McGinnis and Foege (1993) estimated that 40% of US deaths were preventable through changing behavior, and this appeared to still be true a decade later (Mokdad et al., 2004). Behavioral treatments can even outperform pharmaceutical treatments, as was the case with the Diabetes Prevention Program (Knowler et al., 2009).

We suggest that several factors have conspired to create a situation where behavioral treatments are not considered worthy of regulatory oversight. First, most behavioral interventions involve commonplace behaviors. In the case of dieting, the behavior is undertaken frequently to improve one’s appearance. Second, the changes in health outcomes are often slow. Most dieting interventions lead to 1 pound per week of weight loss, whereas, for example, a dose of epinephrine will immediately cure anaphylaxis. Third, behavioral interventions are not perceived to carry the same level of risks as pharmaceutical treatments – they are not ingested, and they are non-invasive. These reasons are certainly understandable, but are fallacious as arguments for why behavioral treatments do not need to be evaluated as rigorously as are pharmaceutical drugs. Commonplace behaviors can be deadly (e.g., driving a car). Health outcomes that change slowly can also be deadly (e.g., cancer). Behavioral interventions may carry fewer risks than pharmaceutical treatments, but they are likely not without *any* risks – risks that do not undergo regulatory oversight before being recommended to patients. Accordingly, in this article we demonstrate how one might evaluate behavioral treatments using the Manual of Policies and Procedures (MAPP) created by the FDA’s Center for Drug Evaluation and Research (2010).

## FDA FRAMEWORK

To obtain FDA approval, five questions must be addressed over the course of four phases of trials. Phase 1 comprises two questions: (1) Is the treatment safe? (2) How dangerous is the condition? The latter question is necessary because the riskiness of treatments must be balanced by the severity of the problems being treated. The more severe the problem, the more we may be willing to use treatments that have potentially harmful side effects. Treatments that involve high risk would not be offered for a common cold, but might be offered for a deadly type of cancer. In Phase 2, the FDA asks: is the treatment effective? In Phase 3, the FDA asks: is the treatment safe and effective for large numbers of people? Phase 4 is a post-approval phase that we will not discuss here.

The most rigorous form of evaluation study is a randomized controlled trial (RCT), and enough of these types of trials exist in eating behavior interventions that we suggest only considering RCTs when applying FDA criteria to them in the context of efficacy. When looking at evidence from clinical trials, the FDA considers the type and sample size of those trials. Phase 1 clinical trials have the smallest sample size and test for the safety of the treatment, and determine whether the treatment has side effects. Phase 2 trials primarily use RCTs of a few 100 participants to test how well the treatment works. Phase 3 trials tend to involve many thousands of people and gather further information about the populations in which the treatment is effective. They may also compare the treatment’s efficacy against the current gold-standard treatment.

## APPLYING FDA CRITERIA TO DIETING

In the following sections, we use dieting as a test case to describe how we might apply the FDA criteria to eating behavior interventions.

### PHASE 1: SAFETY OF TREATMENT VERSUS DANGEROUSNESS OF CONDITION

#### Is dieting safe?

Any drug, in order to be approved by the FDA, must be evaluated for its safety. All the side effects of the drug must be reported so that we may ultimately assess whether the benefits of the treatment outweigh those risks. Examples of “side effects” in the dieting context might be eating disorder pathology (Stice et al., 2011), binge eating (Polivy and Herman, 2002; Wadden et al., 2004), malnutrition (French and Jeffery, 1994), smoking, alcohol, and marijuana use (Krahn et al., 2005; Crow et al., 2006; Seo and Jiang, 2009), depression (Wadden et al., 2004), psychological distress (Isomaa et al., 2010), lower self-esteem (Pesa, 1999; Ackard et al., 2002), cognitive deficits (McFarlane et al., 1999), weight cycling (Diaz et al., 2005), and stress (Tomiyama et al., 2010).

In discussing side effects, several considerations should be addressed. Were the side effects observed in studies with longitudinal or cross-sectional designs, rather than designs that allow for causal inference? Some judgment must be made as to how to balance studies documenting the respective side effects countered by studies failing to observe them. The timeframe of studies varies widely, and it is unclear whether studies that test for but do not find evidence of specific side effects have sufficiently long follow-up periods to observe the risks of dieting. Alternately, and particularly in the case of experimental laboratory studies that allow for causal inference but take place within a timeframe of hours to days, documented side effects may be short-lived and of minimal concern.

#### How dangerous is the condition?

This question seems straightforward, and often is in the case of pharmaceutical treatments. In the context of behavioral treatments, however, this question becomes more complex, as there are multiple “conditions” that behavioral treatments are meant to affect. Even in the case of dieting treatments, where the condition that dieting is meant to treat is almost always overweight and obesity, the answer to this question is unclear. Although the American Medical Association recently voted to label obesity as a disease, there is substantial evidence that being overweight does *not *increase risk for mortality (Flegal et al., 2013) or disability (Alley and Chang, 2007), and the mortality risks associated with obesity appear to have decreased over time (Flegal et al., 2005). Furthermore, the majority of obesity-related deaths occur among individuals with a body mass index (BMI) 35 and above (Flegal et al., 2013). The research clearly shows that overweight is not consistently associated with disability or mortality and that many overweight and obese individuals experience few, if any, adverse health consequences due to their weight. In many studies, obesity is in fact associated with *better* outcomes – a phenomenon known as the “obesity paradox” (Clark et al., 2011). These results may indicate that the true problem is with the current system of using BMI categories to characterize excess weight (Kuczmarski and Flegal, 2000). Nevertheless, if overweight and obesity under current BMI categories and guidelines are the “conditions” that the FDA must balance against the risks of dieting, the risks will need to be shown as extremely minimal at BMI ranges below 35.

Other conditions that dieting interventions are meant to treat include hypertension, hypercholesterolemia, or insulin resistance. The evidence that these conditions are dangerous is much more defensible (see Tomiyama et al., 2013 for a review). Some individuals diet to improve their quality of life and reduce disability, rather than to reduce their risk of death or disease. And indeed, Jia and Lubetkin (2010) reported that all classes of obesity combined contributed to 0.05 quality-adjusted life years lost; a number that has more than doubled since 1993. However, the FDA does not include quality-adjusted years of life lost in their consideration for approval, and as we summarize next, the criterion on which dieting interventions are currently evaluated is, in the overwhelming majority of trials, weight change.

### PHASES 2 AND 3: EFFICACY

#### What does it mean for dieting to work?

***What to measure***. In order to assess whether dieting is an effective treatment for overweight and obesity, we must first decide on appropriate criteria upon which to judge it. The MAPP refers to this criterion as the primary endpoint, and for dieting, this endpoint is nearly always weight loss. Although we would argue that improved health rather than weight loss should be the measure of effectiveness, we recognize that weight loss is the currently accepted definition of success. We therefore summarize how FDA Phase 2 criteria would be applied when evaluating the effectiveness of dieting in reducing weight.

The necessary amount of weight loss for a diet to be considered effective is somewhat arbitrary, and it has changed dramatically since researchers first started routinely studied dieting. The original standard for success required dieters to reach a “normal” weight as defined by the Metropolitan Life Insurance Company (1942). For example, an average height woman (5′5′′) of medium body frame was expected to weigh about 134 pounds (Metropolitan Life Insurance Company, 1942), so a 200-pound woman of average height would need to lose 66 pounds to be considered a successful dieter. This standard was rarely achieved (Stunkard and McLaren-Hume, 1959), and over the next 50 years the standard changed from losing 20% of one’s starting weight, to 10%, and to just 5% of one’s starting weight (Institute of Medicine, 1995, p. 5). Now, an average height woman weighing 200 pounds needs to lose just 10 pounds to be considered a successful dieter. The use of this criterion as the primary endpoint would need to be justified to the FDA in terms of its validity and whether it provides “a reasonable assessment of clinical benefit” (Center for Drug Evaluation and Research, 2010).

***When to measure it***. In addition to selecting an appropriate endpoint, researchers also need to decide when that endpoint should be assessed. The current standard set by the Institute of Medicine is that individuals need to maintain a 5% weight loss for a year. The year, however, is counted as beginning when the diet *begins*, rather than beginning when that target weight is reached. This convention is likely used to make it easier to evaluate and compare diets, because individuals reach target weights at different time-points. Regardless, the 1 year weight loss maintenance standard is not actually a measure of whether one maintained weight loss for a full year, but rather whether an individual is at the target weight 1 year after beginning the diet. The MAPP requires a discussion of the “adequacy of duration” of the clinical trials, and it is not clear if this maintenance period would be considered adequate.

The issue of when to judge the success of a treatment is particularly complex when it comes to dieting. Dieters tend to take off weight quickly at first, then more slowly, followed by weight regain over several more years (Garner and Wooley, 1991). The effectiveness of the diet will look quite different if it is measured at the end of the early stage when weight has come off, or later, as it is regained.

Pharmaceutical treatments differ on whether they are evaluated during active treatment or at some time point after treatment ends. An antibiotic would not be considered effective if the bacteria were only eradicated during the time individuals were taking the medication, and then came back again after the treatment ended. Similarly, chemotherapy for cancer would not be judged effective if tumors reappeared immediately at the end of treatment. On the other hand, medications such as anti-depressants and pain medications are only expected to be effective while individuals are taking them.

It can be argued that diets are only expected to “work” while individuals are actively engaging in them. If so, then the short-term effectiveness would be considered the appropriate measure. Proponents of this viewpoint argue that diets would work if individuals would just stay on them, and the short-term effectiveness is the only measure taken while individuals are still restricting their intake. It is not yet known, however, if diets stop leading to weight loss because individuals stop restricting their eating, or if individuals stop restricting their eating because the diets stopped leading to weight loss (or a combination of these). There is evidence that dieters’ weight loss tends to level off during diets even while they adhere to the diet (Tataranni and Ravussin, 2004), and that individuals who successfully lose weight at a certain calorie level cannot necessarily *maintain *the new weight at that calorie level (Leibel and Hirsch, 1984). If this is the case, then dieters should be informed that they can only expect their diet to work for a somewhat brief length of time. This is not a unique circumstance. For example, extended use of medications such as alprazolam (Xanax) and diazepam (Valium) can result in medication tolerance (i.e., requiring higher doses to achieve the same effect; Ellinwood et al., 1985), and these anxiety medications are usually explicitly recommended for short-term use. Another consideration is whether the use of dieting treatment might make subsequent dieting treatments less effective (Tataranni and Ravussin, 2004), just as cochlear implants can render other treatments for hearing loss impossible (Lenarz et al., 2013).

In terms of evaluating the effectiveness of diets, we are proponents of the viewpoint that one cannot consider a diet successful if individuals rapidly regain the weight they lost. According to this viewpoint, the longer-term effectiveness is the appropriate measure. Obesity-related illnesses tend to be chronic diseases, such as cardiovascular disease and diabetes, and to be successful, we believe their treatments must lead to long-term benefits.

***Study design***. In addition to describing the outcomes of the clinical trials, the MAPP also requires a discussion of aspects of study design that might limit the conclusions that can be drawn. One area of focus is whether the validity of the study is threatened by “subject disposition,” which includes both the rate of subject exclusion from entry into the study and the rate of drop-outs.

Generalizability can be threatened when the exclusion criteria for a study are so extensive that the sample is not representative of typical users of that drug. In dieting studies, participants can be excluded if their BMI is above a certain cut-off, they have a present or past diagnosis of heart disease, angina, or other physical or psychological illnesses, or if their levels of certain macro-or micro-nutrients (e.g., glucose, sodium) are too high (reviewed in Tomiyama et al., 2013). These exclusions limit the generalizability of the findings to just healthier dieters. Of more concern in terms of generalizability, several studies exclude subjects that researchers felt would not be able to adhere to the study requirements. These multiple enrollment steps, called “run-in periods,” are often recommended in clinical trials and are intentionally stringent in order to isolate a participant pool that will remain in the study until the end (Friedman et al., 2010). This may result in samples of participants who are more motivated and more successful at altering their behavior than the average dieter. In one study, for example, 18.8% of the potential participants were excluded before the study began for failing to control their diabetes with their diet for the previous 6 weeks (Hanefeld et al., 1991).

Generalizability can also be threatened due to study attrition (for a more detailed analysis of this problem, see Mann et al., 2007). Many reasons for loss to follow-up are seemingly random (e.g., the subject moves and can no longer be located by the researchers). Others could be problematic (e.g., excluding subjects from analyses because of serious illness or death). To the extent that these drop-outs occur in differing proportions among the diet and control conditions, internal validity may also be threatened.

### PHASE 3: THE ISSUE OF ADHERENCE

A starting point to determine the patient populations for whom dieting should be approved is examining the exclusion criteria for the original trials. Adherence to dieting is the most consistent predictor of weight loss and changes in health outcomes (Pagoto and Appelhans, 2013), and as noted above, non-adherent individuals are often excluded from trials. Despite the importance of adherence, dieting interventions are not evaluated based on whether participants are able to adhere to the regimen. Adherence is often raised as the reason that a given trial succeeded or failed, but that is a separate issue from using adherence as a criterion upon which to judge dieting. The FDA addresses the issue of adherence in section 208.1 of its code of regulations, and states that special labeling should be used in cases when “patient adherence to directions for use is crucial to the drug’s effectiveness.” Under an FDA framework, dieting might be prescribed along with label information about the importance of adherence for the treatment’s effectiveness, including information that even with 100% adherence, weight loss may plateau, as noted above.

## DISCUSSION

In this Perspective article, we considered whether viewing behavioral interventions through the lens of FDA regulations is worthwhile. Using the FDA’s framework illuminated areas for future research that may not have otherwise been considered. For example, in evaluating dieting, current researchers do not consider the intervention balanced against the severity of the condition it is designed to treat. Dieting occupies a significant portion of an individual’s resources, and it may also prevent individuals from attempting other forms of treatment. This analysis has also uncovered that more research is needed on efficacy in relation to whether weight is lost but then regained; the length of treatment that is required for a given level of efficacy; how consistently and with what level of adherence the dieting treatment must be “applied” to reach efficacy; and whether adherence to the treatment is a realistic expectation. A final strength of applying FDA criteria to dieting is that it can serve to refocus the goals of dieting trials squarely on improving health rather than reducing weight. Incorporating this framework into traditional methods of evaluation may lead to new insights in the search for effective behavioral treatments.

## Conflict of Interest Statement

The authors declare that the research was conducted in the absence of any commercial or financial relationships that could be construed as a potential conflict of interest.
